# Correlation between the clinically diagnosed inflammatory process and periapical index scores in severely painful endodontically involved teeth

**DOI:** 10.1111/iej.13407

**Published:** 2020-10-13

**Authors:** D. K. Rechenberg, A. Munir, M. Zehnder

**Affiliations:** ^1^ Division of Endodontology Clinic of Conservative and Preventive Dentistry Center of Dental Medicine University of Zurich Zurich Switzerland

**Keywords:** apical periodontitis, dental radiology, PAI, pain, pulpitis

## Abstract

**Aim:**

To assess and correlate three distinct states of severely painful endodontically derived inflammation with their depiction on periapical radiographs using periapical index (PAI) scores.

**Methodology:**

During a period of 15 months, 368 consecutively enrolled patients with suspected endodontic emergency conditions were examined at the University of Zurich, Center of Dental Medicine. Cases with a severely painful (numeric rating scale, NRS‐11 > 6) endodontically involved tooth and a clear pulpal and apical diagnosis (*n* = 162) were selected (one tooth per patient). Teeth were divided into three groups according to the clinically diagnosed main location of the inflammatory process: level 1: pulp (positive response to cold test), level 2: periodontium (no response to cold without swelling) and level 3: periapical tissues (no response to cold with swelling). Periapical radiographs were obtained using a digital unit and analysed by two calibrated observers. For level 2, which had the highest PAI variance (*n* = 76), the PAI scores were further scrutinized regarding their dependence on tooth location and the duration of pain. Data were analysed using chi‐squared and non‐parametric tests, alpha = 0.05.

**Results:**

Overall, the PAI scores correlated well with the clinically diagnosed main location of periapical inflammation (Spearman’s rho = 0.5131, *P* < 0.001), with level 1 having the lowest scores by far (*P* < 0.001) and level 2 having significantly lower scores compared to level 3 (*P* < 0.05). However, a PAI score of 5 was found in merely 3 teeth within the entire cohort, and 49% of the teeth in the level 2 group had no radiolucency (PAI < 3). Within level 2, the PAI scores were not dependent on tooth location but were substantially (*P* < 0.001) higher for teeth which had hurt for more than one week, and for root filled teeth.

**Conclusions:**

For the analysed, severely painful endodontically involved teeth, the clinically diagnosed main location of inflammation was reflected by the periapical index. PAI scores were not significantly influenced by anatomical noise, yet in some cases under‐estimated the clinical situation.

## Introduction

Endodontic diagnosis remains a debated issue. In the past, histological and clinical/radiographic terms were mixed, and a correlation between these terms and the true state of the tooth was low (Baume 1970, Dummer *et al*. 1980). In an approximation to a classical paper by Morse and co‐workers (Morse *et al*. 1977), an attempt was made to avoid such problems and use simple terms based on patient history, clinical signs and symptoms and radiology (American Association of Endodontists 2009, Glickman 2009). Given the biological fact that endodontic lesions originate in the pulp space and then, at a later stage, can progress towards the periapical tissues (Langeland 1987), this nomenclature by the American Association of Endodontists (AAE) is based on pulp sensitivity tests and periapical tests in the form of tenderness to percussion and palpation. Whilst these newer terms, especially the concept of ‘reversible’ versus ‘irreversible’ pulpitis, remain doubtful in non‐painful teeth (Hasler & Mitchell 1970), the correlation between clinical and histological findings is much clearer in teeth associated with spontaneous pain (Ricucci *et al*. 2014), and severely painful teeth are easy to diagnose (McCarthy *et al*. 2010). However, one aspect in this context has not been studied in detail and that is the appearance of painful endodontic conditions on periapical radiographs. From a clinical, diagnostic point of view, this may seem negligible, since in such cases an endodontic intervention is indicated whether a rarefaction is seen on the radiograph or not. However, considering the biological foundation of endodontic disease, the relevance becomes obvious. The nociception of teeth and their surrounding oral structures dictate that inflammation of different tissues can, but does not have to, elicit pain (Byers & Narhi 1999). These tissues are (i) the pulp itself, when it is highly inflamed, (ii) the periodontal ligament and (iii) the periosteum once the inflammation has reached beyond the periapex.

Periapical radiographs have traditionally been used as the core surrogate outcome to study the course of healing in endodontically treated teeth (Ørstavik 1996). Based on a histological observation of teeth in human cadavers and their display on periapical radiographs (Brynolf 1967), an index was developed that depicts 5 stages of periapical inflammatory changes, from not present to highly inflamed (Ørstavik *et al*. 1986). This index was termed the periapical index (PAI) scoring system. Besides its source, the PAI system has another unique feature: it is based on visual references in the form of corresponding drawings depicting typical histological stages as they appear on periapical radiographs (Brynolf 1967, Ørstavik *et al*. 1986). However, it must be conceded that the observations underlying the PAI were obtained from maxillary anterior teeth (Brynolf 1967), and the anatomical noise due to superimposition of overlying structures that is associated with periapical radiographs on posterior teeth was not addressed (Cheung *et al*. 2013). Since the Brynolf (1967) observations were made on human cadavers, there was no way of finding out whether the studied teeth were symptomatic at the time of death.

Over the past decade, three‐dimensional radiographic imaging in the form of cone‐beam computed tomography (CBCT) has become widely available. This technology, however, is not recommended for routine cases because of its inherently high radiation dosage (Pauwels *et al*. 2012, ESE 2019). Nevertheless, recent studies using CBCT imaging have shown that periapical changes can already be detected in teeth with inflamed vital pulps and that these changes can be transient (Hashem *et al*. 2015). This is in line with a study targeting mediators for bone resorption (RANKL/OPG) and inflammation (IL‐8) in pulpal and periapical disease. Data from this study suggest that bone resorption precedes inflammation (Rechenberg *et al*. 2014). In two‐dimensional radiography, however, such changes associated with inflamed vital pulps have not been examined, even though the idea that smaller lesions cannot be detected on periapical radiographs of posterior teeth (Bender & Seltzer 1961a, 1961b) has recently been challenged (Chang *et al*. 2020).

Because two‐dimensional radiography still is a core pillar in endodontic diagnosis, how inflammatory changes in bone can be depicted on these images remains a relevant topic. Surprisingly, there appears to be a clear lack in data linking highly painful inflammatory endodontic conditions to periapical radiographs. In this study, three clinical conditions representing three distinct stages in the progress of endodontically derived inflammation (and suspected extent of bone resorption) were correlated with their appearance on periapical radiographs using the PAI scoring system in adult patients recruited from the emergency unit of the dental school at the University of Zurich. The three clinical conditions were as follows: painful tooth still responding to cold test (main location of inflammation: pulp, level 1), painful tooth not responding to cold test without swelling (main location of inflammation: periodontal ligament, level 2) and painful tooth not responding to cold test with swelling (main location of inflammation: periapical tissues, level 3). In addition, the PAI was scrutinized in the intermediate group (level 2) regarding its dependence on tooth types (molars versus other teeth), the jaw the tooth was located in and the dynamics of the painful episode.

## Methods

### Study design and ethics

This observational study was performed in accordance with the Declaration of Helsinki, and the protocol was approved by the local ethics commission. The reporting follows the STROBE guidelines for reporting of observational studies. Emergency patients attending for a non‐scheduled visit to the emergency unit of the Clinic of Conservative and Preventive Dentistry, Center of Dental Medicine, University of Zurich, with a suspected painful endodontic condition were screened consecutively over a period of 15 months. The patients were asked about their main complaints and symptoms; then, the clinical case presentation was assessed by the dentist on call, supervised by one of the authors (D.K.R.), who is a senior endodontist and head of clinic. Only adult patients 18 years and older participated in the study. Written informed consent was obtained from all participants.

### Exclusion criteria

Patients were not included if they refused to participate, were pregnant (i.e. could not be examined radiographically), were on immunosuppressant therapy, were on long‐term anti‐inflammatory drugs or took antibiotics during the previous month. The aim was to exclusively include patients with severely painful endodontic conditions. Therefore, patients presenting without severe pain (NRS‐11 score < 7) were excluded. Cases were also excluded if dental emergency treatment had already been initiated elsewhere during the painful episode, or if the endodontic diagnosis could not be established reliably. Moreover, cases with periapical radiographs not meeting the quality criteria required for a PAI evaluation were excluded as well. For all patients, whether included or excluded from the study, appropriate therapy was carried out after the assessment.

### Clinical and radiographic examinations

The evaluation included a comprehensive case history including the quantification of the maximum pain intensity perceived within the last 24 h rated on a numeric rating scale (NRS‐11; (Warrant Grant Magnusson Center 2003). ‘No pain’/’pain free’ (NRS = ‘0’) and ‘worst pain imaginable’ (NRS = ‘10’) were set as anchors. Patients were also asked about the pain history of the involved tooth, that is whether it had hurt spontaneously for more or less than one week. The detailed clinical examination included visual inspection of the pain‐causing tooth and corresponding soft tissues, pulp testing with carbon dioxide snow, tenderness to percussion, probing depth and evaluation of tooth mobility. The examinations were always compared to the neighbouring and/or contralateral teeth free of any complaint. All relevant information was recorded in a separate chart. Subsequently, periapical radiographs were made using a film holder in paralleling technique and a beam‐aiming radiographic unit (Heliodent; Sirona, Bensheim, Germany) operating at 60 kV and 7 mA. Photostimulable phosphor plates (Digora Optime; Soredex, Tuusula, Finland) were used as receptors. The exposure time ranged between 0.2 ms (front teeth) and 0.25–0.32 ms (posterior teeth). The radiographs were evaluated on a display designed for visualization of digital radiographic images (MDview 243; NEC Corporation, Tokyo, Japan).

### Data collection

Patient‐related data (gender and age) and relevant case/tooth‐related data including the World Dental Federation notation of the affected tooth, tooth group (incisor, canine, premolar and molar), location (maxilla and mandible), type of restoration (none, direct filling, crown/indirect restoration and bridge abutment), presence of root filling (y, n), tenderness to percussion (y, n), positive response to pulp sensitivity test (y, n) presence of swelling (y, n), and the pain level (NRS‐11) and duration were tabulated in a standardized form.

### Periapical index

Periapical health was evaluated radiographically using the periapical index scoring system (Ørstavik *et al*. 1986) with its 5‐point radiographic reference scale to score the absence, presence and increasing severity of periapical disease. Two examiners (A.M. and D.K.R.) were calibrated against a set of 100 standard radiographs pre‐scored by the developers of the index. Disagreements were resolved by discussion. The calibration was recorded twice within a 2‐week interval, and inter‐ and intra‐observer agreements (weighted kappa values) were calculated (see below). After calibration, the periapical radiographs of the teeth under investigation were evaluated under standardized conditions by both examiners. For multirooted teeth, the highest of the PAI scores allocated to the individual roots was used. In case of uncertainty, the higher of the scores was assigned. Again, disagreements were resolved by discussion. The examiners were blinded to the identity and therefore to the clinical condition of the cases they were assessing.

### Data analysis and statistics

Kappa statistics were used to assess intra‐ and inter‐observer agreement (Landis & Koch 1977). For the statistical analysis, the cases were grouped according to their clinically diagnosed main location of the inflammatory process: level 1: pulp (positive response to cold test), level 2: periodontium (no response to cold without swelling) and level 3: periapical tissues (no response to cold with swelling). The correlation between these three stages of clinical progress of inflammation and PAI scores was assessed by computing the Spearman correlation coefficient. Ordinal data sets such as the PAI scores were compared between groups using Pearson’s chi‐squared and Fisher’s exact tests (for dichotomous variables). Continuous data sets were compared using parametric statistics. Bonferroni correction was applied for multiple testing. The alpha‐type error was set at 5% (*P* < 0.05).

## Results

### Recruitment and demographics

**Figure 1:**
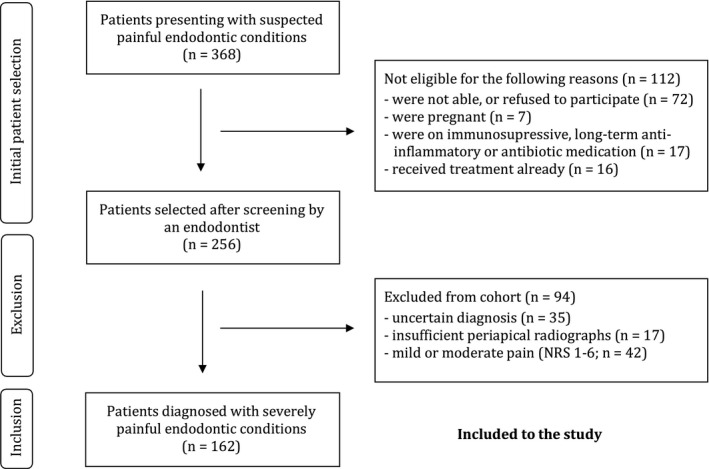
Flowchart depicting patient selection.

Over a period of 15 months, 368 patients suspected of having a painful endodontic condition presented at the emergency unit of the Clinic of Conservative and Preventive Dentistry and were further evaluated to participate in the study. Of the 368 patients, two hundred fifty‐six (*n* = 256) had painful endodontic conditions confirmed by a dentist specialized in endodontics and could be recruited for participation (Fig. 1). Subsequently, ninety‐four patients (*n* = 94) had to be excluded from the analysis with reasons (Fig. 1). Finally, 162 patients having a tooth with a severely painful endodontic condition could be included to the study. The mean age of these included patients was 40 ± 15 years. The gender ratio was 61 females to 101 males. Most of these individuals had a tooth that was negative to cold but did not have a periapical swelling (Table 1). The pain history of the teeth that still tested positive to cold was significantly (*P* < 0.05) longer than that of cold‐negative counterparts (Table 1). Additionally, in this group of teeth that were still sensible to cold there were significantly more molars (*P* < 0.05) and fewer restored teeth than in the groups that tested negative to the cold test (Table 1).

**Table 1:** Patient‐ and tooth‐specific findings according to clinical presentation of the severely painful endodontically involved teeth under investigation and their clinically diagnosed level of inflammation spread (*n* = 162)

	Cold‐positive Level 1	Cold‐negative, no swelling Level 2	Cold‐negative, swelling Level 3
Total counts (*n*)	55	76	31
Gender (f/m)	18/37	31/45	12/19
Age^a^ (years ± SD)	36 ± 12^A^	41 ± 15^AB^	45 ± 15^B^
Pain duration^a^ (<1 week/ >1 Week)	23/32^A^	55/21^B^	21/10^B^
Percussion (pos/neg)	50/5	70/6	29/2
Jaw (maxilla/mandible)	27/28	28/48	14/17
Molar tooth^a^ (yes/no)	45/10^A^	50/26^AB^	15/16^B^
Type of restoration^a^ (none/fi/cr/ab)	22/32/1/0^A^	22/40/9/5^B^	12/11/4/4^B^
Root filled^a^ (yes/no)	0/55^A^	9/67^B^	8/23^B^

ab, bridge abutment; cr, crown/indirect restoration; f, female; fi, direct filling; ft, front tooth; m, male; m, molar; neg, negative; pm, premolar; pos, positive; SD, standard deviation.

^a^ When there were significant overall differences between the clinical presentations (chi‐squared test or ANOVA), individual data sets that did not differ (*P *> 0.05) from each other in the *post hoc* analysis are marked with an identical superscript letter (Fisher’s exact test/chi‐square test or *t*‐test, Bonferroni correction).

### PAI scoring observer agreement

Observer calibration resulted in weighted kappa values of 0.64 (A.M.), 0.72 (D.K.R) and 0.61 for intra‐ and inter‐observer agreement, respectively, suggesting ‘substantial agreement’ (Landis & Koch 1977).

### Correlation between clinical and radiological findings

**Figure 2:**
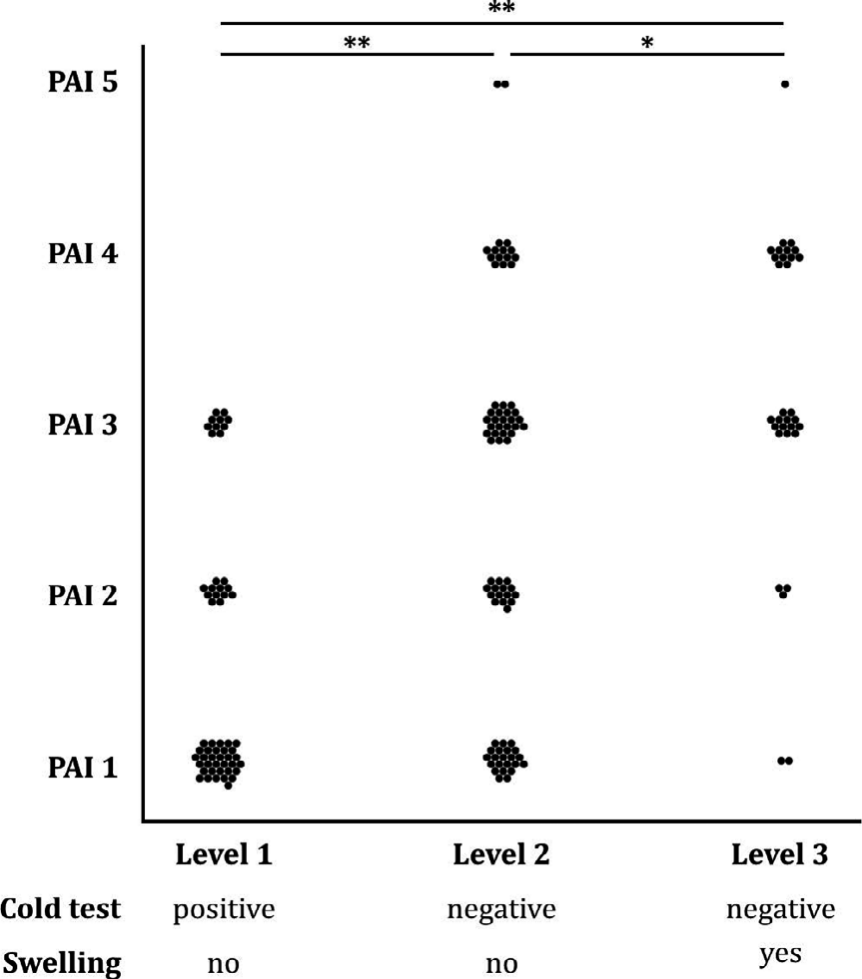
Dot plot showing the correlation between the clinically diagnosed main location of the painful inflammation and PAI scores (Spearman’s rho = 0.5131, *P* < 0.001). Level 1: pulp, level 2: periodontium and level 3: periapical tissues. Each dot represents one case/tooth. A double asterisk denotes a highly significant difference between two data sets (chi‐squared test, *P* < 0.001), a single asterisk a significant difference (*P* < 0.05).

A good correlation (Spearman’s rho = 0.5131, *P* < 0.001) was found between main location of the painful inflammation and the PAI scores of the periapical radiographs of the affected teeth (Fig. 2). The lowest PAI values were given to the teeth that were still positive to the cold test (*P* < 0.001 against both groups that responded negative to the cold test). Furthermore, amongst the teeth that responded negative to the cold test, the PAI scores of those teeth with a periapical swelling (level 3) were significantly higher than PAI scores of teeth without swelling (level 2, *P* < 0.05). Moreover, if a PAI score of 3 and higher is considered to represent a periapical rarefaction, then 37 of the 76 teeth (49%) with negative cold test and no periapical swelling and 5 of the 31 (16%) teeth with periapical swelling showed no radiological signs of bone resorption on the day of the emergency visit. Merely 3 teeth, 2 in the cold‐negative group without swelling and 1 in the group with periapical swelling, had a PAI score of 5 (Fig. 2).

### Further assessment of the PAI

To assess the PAI scoring system further, the largest reference group of teeth in this study was used: non‐sensible to cold without swelling (level 2, *n* = 76). This was done because the PAI scores had the highest range in that clinical condition (Fig. 2). It was checked whether higher PAI scores were found with teeth associated with less anatomical noise on periapical radiographs, that is non‐molars versus molars and teeth in the mandible versus the maxilla (Table 2). This was not the case, as PAI scores between these groups did not differ significantly (*P *> 0.05). On the other hand, clinical parameters that were likely to be positively associated with inflammatory bone resorption in this group did result in higher PAI scores at a highly significant level (*P* < 0.001): teeth with a pain history of more than one week and root filled teeth had higher PAI scores than teeth that had not been painful for this duration or were not root filled, respectively.

**Table 2:** Association of the PAI score of the severely painful endodontically involved teeth that were negative to the cold test and had no swelling (level 2 clinically diagnosed level/spread of inflammation, *n* = 76) to variables that can be related to the extent of inflammatory bone resorption^a^ or anatomical noise on the periapical radiograph^b^

Independent variable	Higher PAI scores with	*P*‐value*
Pain history (<1 week/ >1 week)^a^	Longer history	0.0008
Jaw (maxilla/mandible)^b^	–	0.0775
Molar (yes/no)^b^	–	0.5366
Root filled (yes/no)^a^	Root filled teeth	<0.0001

* Pearson’s chi‐squared test.

## Discussion

This study, performed on teeth with severely painful endodontic conditions of consecutively enrolled dental emergency patients, revealed that digital periapical radiographs assessed using the PAI scoring system correlated well to the clinical situation. PAI scores reflected the clinically suspected progression of inflammation from within the pulp space to the periodontal ligament and then the periapical tissues. Moreover, the PAI score in these teeth was not significantly influenced by anatomical noise. However, many of the painful teeth that contained a clinically non‐vital pulp did not show any signs of radiological rarefaction at the time of assessment.

To appreciate the current results, it should be acknowledged that, despite a certain degree of uncertainty in endodontic diagnostics, the continuum on which the cascade of clinical symptoms assessed here can be divided into 3 distinct, but progressive, subsets: 1) pulpal pain, 2) spontaneous pain in a tooth containing a necrotic or necrotizing pulp deriving from the periodontium and 3) pain in the periapical tissues with visible clinical evidence of pus or inflammatory exudate. Endodontic treatment decisions are made based on a combination of clinical and radiographic findings. Considering the fact that periapical radiographs are routinely used in endodontic diagnosis, it is surprising that the appearance of these severely painful endodontic conditions on such images has not been studied in a standardized manner in living humans. This was attempted in this study; however, there were no histological or three‐dimensional radiographic controls to the findings that are presented here. Histology was not possible for obvious ethical reasons. Cone‐beam computed tomography (CBCT) scans of each of these emergency cases would not have been allowed by the local ethical review board, due to unnecessary exposure to ionizing radiation in such easily diagnosable cases (Dula *et al*. 2015). Nevertheless, correlative studies between CBCT and periapical radiographs performed in routine endodontic recall cases unequivocally showed that periapical radiographs underestimate periapical lesions (Cheung *et al*. 2013). On the other hand, a significant information gain by using CBCT imaging has not always been shown to be evident (Kruse *et al*. 2015). Diagnosis made using CBCT images is associated with a reduced specificity in identifying periapical inflammatory lesions, *that is* rarefactions may be detected on CBCT images of teeth that are actually not diseased (Pope *et al*. 2014). The digital radiographic system used here had a resolving power of 14.3 lp mm^−1^ at a resolution of 35 µm. This compares favourably to the 80–100 µm voxel size achieved using state‐of‐the‐art small field‐of‐view CBCT unit (McGuigan *et al*. 2020). Moreover, in contrast to what Cheung and co‐workers found in their study (Cheung *et al*. 2013), anatomical noise did not appear to impair PAI scores significantly in the cohort of this study. However, it should be reiterated that this line of argumentation is not meant to disregard the advantages of CBCT imaging in treatment planning of selected complex endodontic cases (ESE 2019).

**Figure 3:**
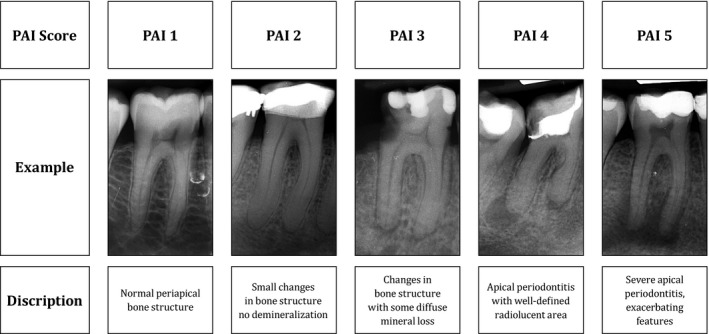
Examples of PAI scores assigned to radiographs of severely painful endodontically involved mandibular molars from the cohort of this study and the verbal descriptors of the PAI score according to Kirkevang *et al*. (2015), modified from Ørstavik (1996).

The PAI scoring system, in its essence, aims to assess the radiographically visible degree of disorganization of bone around the root apex (Ørstavik *et al*. 1986). The fact that it was based on a classic study that compared the radiographical and histological assessment of teeth in human cadavers (Brynolf 1967) sets it apart from a proposed respective scoring system for CBCT images (Estrela *et al*. 2008). This study suggests that PAI scores can be applied for the assessment of posterior teeth (Fig. 3). The observations reported here confirm an older study, which showed that the apparent width of the periodontal ligament and structure of the periapical trabeculae on periapical radiographs are core features for the assessment of periapical radiographs (Kaffe & Gratt 1988). However, they made no discrimination amongst types of pain or the suspected location of inflammation and thus resulting bone resorption.

The nomenclature for pulpal and periapical conditions defined by the AAE (AAE 2009, Glickman 2009) was avoided in this paper. This nomenclature, based on a paper by Morse and co‐workers (Morse *et al*. 1977), reflects the historic understanding that the term ‘acute’ can only be diagnosed histologically and reflects presence of inflammatory cells. Hence, the term acute was completely avoided in the paper by Morse and co‐workers, and the current AAE terminology uses ‘symptomatic’ versus ‘asymptomatic’. However, unbearable pain forcing a patient to seek emergency treatment from a clinical perspective is completely different from a tooth that is merely sensitive to percussion (Torabinejad & Walton 1991). Inflammatory conditions of endodontically involved teeth can be excruciatingly painful (Rechenberg *et al*. 2016), and lead to out‐of‐hour dental visits and immediate systemic complications (Nalliah *et al*. 2011). The conditions under investigation here were not difficult to diagnose, yet they reflected a clinical succession of the inflammation spreading from the pulp space (pulp still sensible or overly sensible to cold test) to the periodontium and to the periosteum (swelling). The three conditions defined in this communication reflected the main location of the inflammatory infiltrate: in the pulp space, apical periodontium, or in the periapical tissues including the periodontium and oral mucosa. According to the AAE nomenclature, however, all of these teeth would have had an apical diagnosis of ‘symptomatic apical periodontitis’, as almost all of them were sensitive to percussion (Table 1).

In line with more recent publications on this topic, emergency cases with necrotic or root filled pulp spaces outweighed counterparts with vital pulps (Sindet‐Pedersen *et al*. 1985, Rechenberg *et al*. 2016). In this study, this may be explained by the fact that primary caries lesions are consistently declining in Switzerland (Menghini *et al*. 2010), and painful inflammatory conditions deriving from necrotic or insufficiently treated and filled root canal spaces have become relatively more frequent. This was also corroborated by the finding that patients with clinically vital pulps in this cohort were younger and the teeth causing the problem were less restored (Table 1).

Until now, assessments of periapical radiographs using the PAI scoring system in clinical studies have focused on the reliability and repeatability of the scores, yet used surrogate outcomes such as changes in scores over time (Ørstavik 1988). Moreover, PAI scores were frequently dichotomized to ‘healthy’ versus ‘diseased’ with a cut‐off between PAI 2 and 3 to be able to process the data using a logistic regression model (Marending *et al*. 2005). Alternatively, the PAI scores were treated as a continuous (Waltimo *et al*. 2005), which is incorrect from a mathematical perspective. PAI scores by definition are ordinal. As suggested by the data of the present study and discussed above, they can show more than just the presence of a periapical lesion. This was considered in more recent studies, in which an ordinal logistic regression model was used to correlate full‐scale PAI scores to several parameters, including the periapical status (full‐scale PAI score at follow‐up examination) and tooth loss (Kirkevang *et al*. 2015, 2017). It showed that the use of the full‐scale PAI could improve the prognostic assessment of a tooth.

The reason why such a high percentage of the teeth with a negative response to the cold test did not show signs of radiological rarefaction (PAI score < 3) is worth further speculation. The most likely explanation is that this was merely an issue of lesion dynamics. Most patients with a tooth that was negative to the cold test in this cohort stated that their tooth started hurting less than a week ago (Table 1). Radio‐opacity generally depends on the presence of heavy elements in a material. In bone, that is the calcium. The periapical radiographs taken for this study represented a point in the developing pathosis of the affected tooth, at which it became severely painful. The inflammatory resorption of bone by decalcification obviously takes time to become radiographically visible, which is in line with the classic study on monitoring of endodontic treatment outcomes on two‐dimensional radiographs (Ørstavik 1996). The majority of the severely painful periapical lesions under investigation apparently had a rapid onset and no pre‐existing chronic lesion. This is also reflected by the low number of previously root filled teeth in the current study material (Table 1) and the fact that a PAI score of 5 was given only very rarely. The score of 5 represents Brynolf’s main group IV, a clear and most likely pre‐existing lesion with signs of spreading (Brynolf 1967). This finding is biologically interesting and awaits further investigation. It appears to be in line with published material suggesting that severely painful exacerbations of persisting periapical lesions are rare events (Yu *et al*. 2012). Whether or not a root canal infection takes an acute course or stays asymptomatic is influenced by the virulence of the root canal microbiota and the state of the host immune system (Zehnder & Belibasakis 2015). Moreover, as the data of this study suggest, a painful periapical inflammation may also be linked to the volume of root canal space that is available to micro‐organisms. This may explain why root filled teeth were under‐represented in the current cohort. However, the issue of how, when and why teeth cause severe pain due to endodontic infections remains largely unknown and should be studied in more detail in the future, as this represents a clinically important aspect of our profession.

## Conclusions

This study found a good correlation between PAI scores and the clinically diagnosed main location of the painful inflammation. PAI scores were not significantly influenced by anatomical noise, yet in some cases under‐estimated the clinical situation. Taken together, these results can be seen as a further confirmation of the usefulness of the full‐scale PAI score.

## Conflict of interest

The authors have stated explicitly that there are no conflicts of interest in connection with this article.
